# Cryoglobulinemic Vasculitis Manifesting as Rapidly Progressive Glomerulonephritis: A Case Report

**DOI:** 10.7759/cureus.96892

**Published:** 2025-11-15

**Authors:** Sritharan Thivacaren, Mihiran Thanigasalan, Anura Hewageegana, Mohamed Nazar Abdul Latiff, Priyani Amarathunga

**Affiliations:** 1 Nephrology, National Hospital of Sri Lanka, Colombo, LKA; 2 Pathology, Faculty of Medicine, University of Colombo, Colombo, LKA

**Keywords:** cryoglobulinemia, hemodialysis, membranoproliferative pattern, rapidly progressive glomerulonephritis, rituximab

## Abstract

Cryoglobulinemic vasculitis is a small- to medium-vessel vasculitis involving the skin, joints, peripheral nerves, and kidneys, due to deposition of immune complexes containing cryoglobulins. Cryoglobulins are immunoglobulins that precipitate below 37°C and are classified into three types: type I (monoclonal) and types II and III (mixed). We report a rare case of mixed cryoglobulinemia associated with monoclonal gammopathy of renal significance (MGRS) presenting as rapidly progressive glomerulonephritis (RPGN). A 35-year-old man presented with generalized edema, frothy urine, microscopic hematuria, and lower limb numbness for three weeks. Laboratory evaluation revealed elevated serum creatinine (8.5 mg/dL), markedly reduced C4 complement (0.5 mg/dL), positive rheumatoid factor, and an IgM monoclonal band on immunofixation. Bone marrow biopsy showed no abnormal plasma cell proliferation. Renal biopsy demonstrated eosinophilic, periodic acid-Schiff (PAS)-positive globules in capillary loops consistent with cryoglobulin deposits, along with a membranoproliferative pattern. Immunofluorescence showed predominant IgM and kappa light chain deposition. A qualitative cryoglobulin assay was positive, confirming type II mixed cryoglobulinemia. The patient received five sessions of plasma exchange with corticosteroids, intravenous immunoglobulin, and rituximab. He required hemodialysis support until he achieved renal recovery. A follow-up visit showed significant improvement in his proteinuria and renal function. This case underscores the importance of considering cryoglobulinemic vasculitis in the differential diagnosis of RPGN and emphasizes the need for early recognition and prompt immunosuppressive therapy to prevent irreversible renal injury.

## Introduction

Cryoglobulinemia is a rare condition that predominantly affects females compared to males, and the typical age of onset ranges from 40 to 50 years [1]. It involves small to medium vessels in the skin, joints, nerves, and kidneys due to cryoglobulin-containing immune complexes. Cryoglobulins are immunoglobulins in the serum that precipitate at temperatures below 37°C [1]. They were classified into three groups. Type Ⅰ are monoclonal (typically IgM, less commonly IgG or IgA), whereas type Ⅱ and Ⅲ cryoglobulins are mixed. Type Ⅱ is characterized by a monoclonal IgM combined with polyclonal IgG, whereas type Ⅲ is polyclonal (1). Types Ⅱ and Ⅲ are associated with chronic infections and autoimmune diseases.

Isolated proteinuria and hematuria are the common renal manifestations of cryoglobulinemic vasculitis. Here, we present a case of mixed cryoglobulinemia associated with monoclonal gammopathy of renal significance manifested as rapidly progressive glomerulonephritis. It is a very rare kind of presentation.

## Case presentation

A 35-year-old man presented with a three-week history of generalized body swelling and frothy urine. He also reported microscopic hematuria. In addition, he described pain and numbness affecting both lower limbs and the chest wall, associated with reduced urine output. Neuropathy predominantly involves the lower limbs. There was no history of fever, cough, photosensitive rash, oral ulcers, arthritis, dry eyes, or dry mouth. He denied any respiratory or gastrointestinal symptoms and had no history of Raynaud phenomenon. There was no history of renal disease, autoimmune illness, or malignancy. He was not on long-term medications and had no known allergies.

On examination, he was pale with bilateral pitting edema extending to the knees. There was no peripheral cyanosis, no retinal or oral lesions, and no lymphadenopathy. Cardiovascular, respiratory, and abdominal examinations were unremarkable. Neurological examination revealed symmetrical sensory and motor impairment in both lower limbs.

Key laboratory results are summarized in Table 1. On admission, his serum creatinine was 8.85 mg/dL (baseline 1.3 mg/dL), serum sodium 128 mmol/L, and serum albumin 2.8 g/dL. Urinalysis showed 25-30 red cells/high-power field (hpf), 10-15 pus cells/hpf, and proteinuria >200 mg/dL. The urine protein-creatinine ratio was 3.3.

**Table 1:** Key laboratory investigation. FBC: Full blood count, ESR: Erythrocyte sedimentation rate, ANA: Antinuclear antibodies, ANCA: Anti Neutrophilic Cytoplasmic Autoantibody, ASOT: Antistreptolysin O titre

	On admission	After treatment	Normal values
Full blood count			
White blood cells (10^9^/L)	7.97	8.7	4.0 – 10.0 (10^9^/L)
Neutrophils (10^9^/L)	7.31	8.1	2.0 - 7.0 (10^9^/L)
Lymphocytes (10^9^/L)	0.24	0.45	0.8 - 4.0 (10^9^/L)
Hemoglobin (g/dL)	12.2	9.0	11.0- 16.0 ( g/dL)
Platelets (10^9^/L)	191000	400000	150 – 450 (10^9^/L)
Erythrocyte sedimentation rate mm/hour	15	N/A	0-15mm/hour
Lactate dehydrogenase U/L	240	N/A	140-280 U/L
Serum Sodium (millimole/L)	128	146	136 – 145 (millimole/L)
Serum Potassium (millimole/L)	4.4	4.5	3.5 – 5.1 (millimole/L)
Serum Creatinine (mg/dL)	8.5	0.97	0.8-1.2 (mg/dL)
Urine full report			
Pus cells	10-15/hpf	2-4/hpf	0-5/hpf
Red cells	25-30/hpf	Occ/hpf	0-2/hpf
Protein	+200mg/dl	+50mg/dl	Nil
Urine Protein: creatinine	3.3	0.56	< 0.2
Total Protein (g/dL)	4.5	N/A	6.4- 8.3 (g/dL)
Albumin (g/dL)	2.8	3.7	3.5- 5.2 (g/dL)
Globulin (g/dL)	1.7	N/A	2.2 – 4.0 (g/dL)
Serum protein electrophoresis	An abnormal monoclonal band present in the gamma region 0.6g/L		
Serum Immune fixation	Monoclonal band seen is IgM kappa		
Serum free light chains			
Kappa/k light chain (mg/L)	18.4	N/A	6.7-22.4 (mg/L)
Lambda light chain (mg/L)	37.7	N/A	8.3- 27 (mg/L)
k/lambda ratio	2.05	N/A	0.26 - 1.65
Qualitative assay for Cryoglobulins	Positive		
Immunological markers			
Antinuclear antibody	Positive	N/A	Negative
Anti- double stranded deoxyribonucleic acid	Negative	N/A	Negative
C-Antineutrophil cytoplasmic antibody	Negative	N/A	Negative
P -Antineutrophil cytoplasmic antibody	Negative	N/A	Negative
Anti-streptolysin O titrer	37.7IU/ml	N/A	less than 200 IU/ml
RO -52 antibody	Positive	N/A	Negative
Anti-Sjogren's syndrome related to antigen A	Negative	N/A	Negative
Anti-Sjogren's syndrome type B	Negative	N/A	Negative
C3 (mg/dL)	55.2	N/A	80- 178 (mg/dL)
C4 (mg/dL)	0.5	N/A	15-45 (mg/dL)
Rheumatoid factor (U/mL)	58.4 (Positive)	N/A	less than 20 (U/mL)
Virology markers			
Hepatitis B surface Antigen (HBsAg)	Non-reactive	N/A	Non- reactive
Hepatitis C Antigen – Antibody (HCV Ag-Ab)	Non-reactive	N/A	Non- reactive
Ultrasound Abdomen	Lt Kidney -11.7 Bilateral Kidneys show increase echogenicity with blurred corticomedullary demarcation. No Hydronephrosis or hydroureter and no organomegaly.		
High resolution computed tomography chest	No significant abnormality in bilateral lungs		
Skin biopsy	Leukocytoclastic vasculitis		

Serum protein electrophoresis demonstrated an abnormal monoclonal band in the gamma region (0.6 g/L), which was identified on immunofixation as IgM kappa. Complement levels were markedly reduced (C3: 55.2 mg/dL; C4: 0.5 mg/dL). Antinuclear antibody was positive (titer 1:320) with negative anti-double-stranded DNA. Tests for hepatitis B surface antigen and hepatitis C antibody were non-reactive.

**Figure 1:**
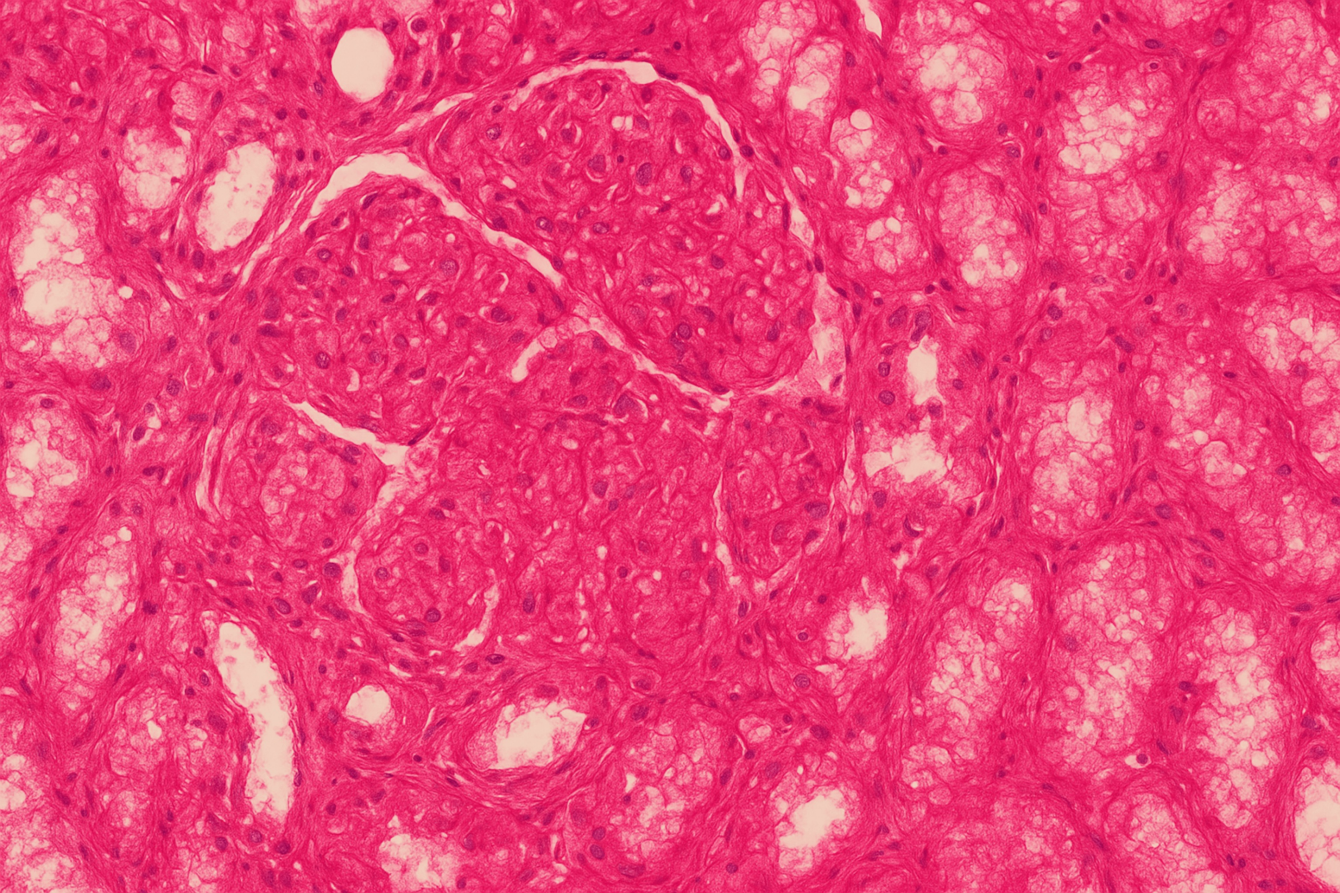
H and E stain into 40 describes a membranoproliferative pattern with eosinophilic globules within the capillary lumen.

**Figure 2:**
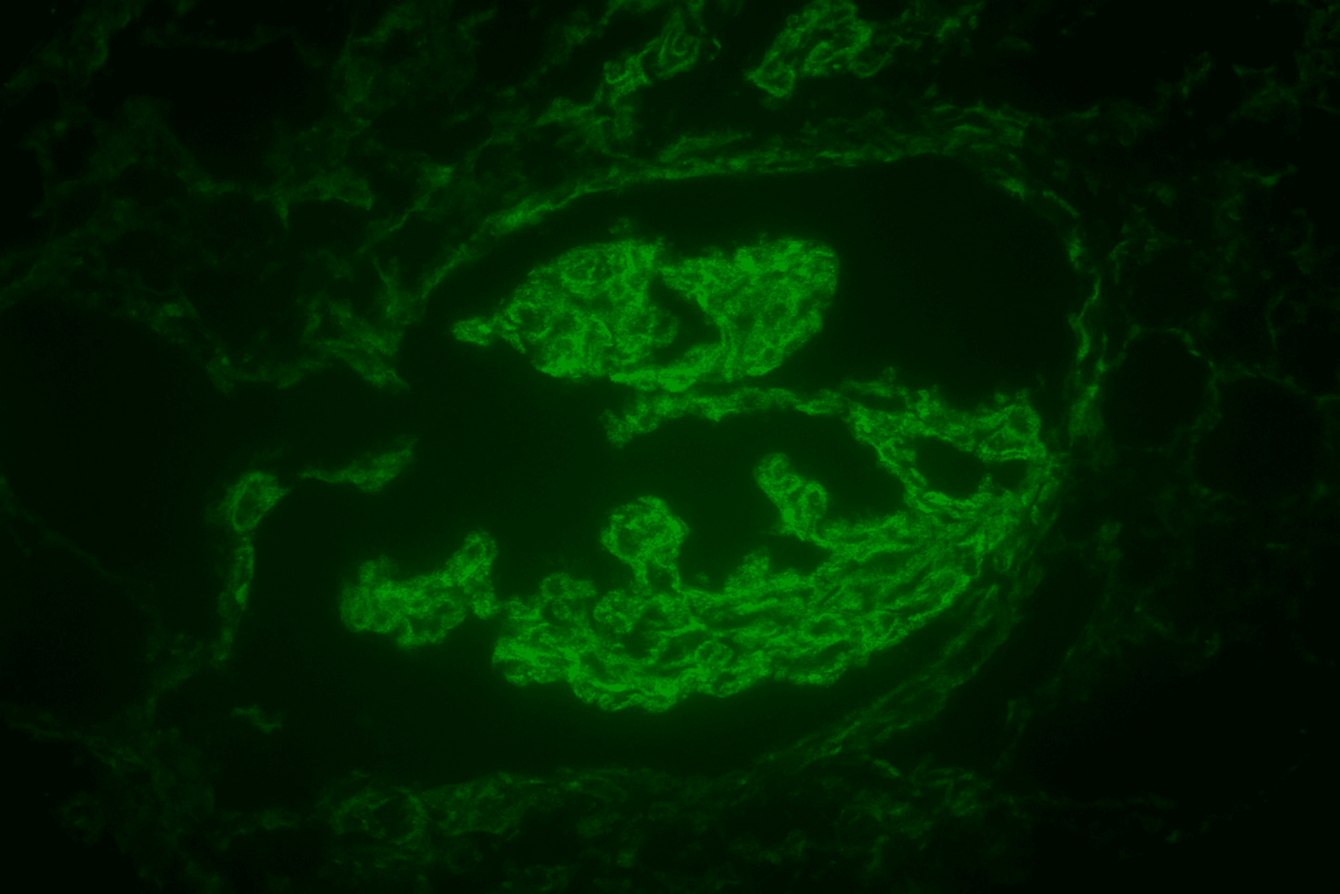
Immunofluorescence staining of mesangium IgM 3+ staining.

**Figure 3:**
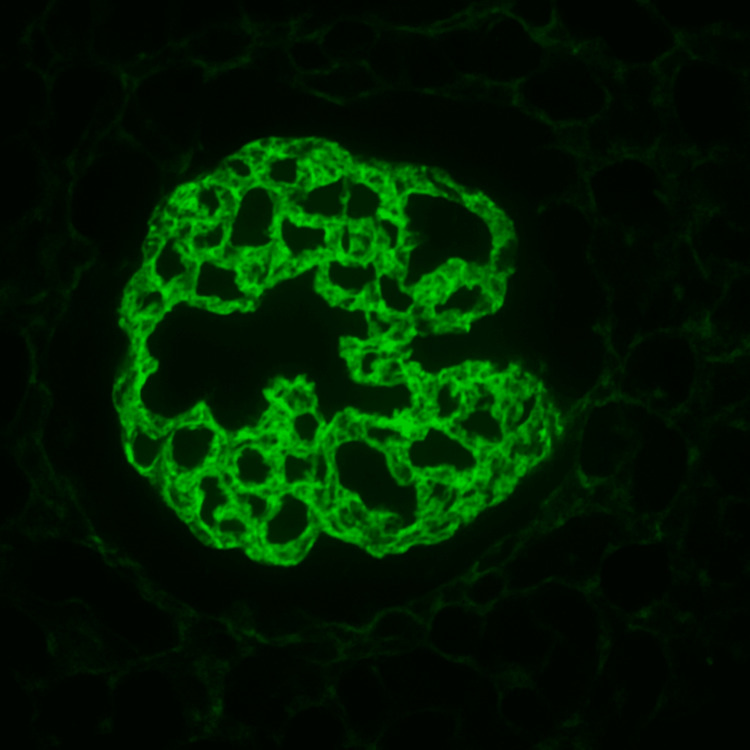
Immunofluorescence staining of mesangium Kappa 3+.

His renal biopsy revealed eosinophilic, deeply PAS-positive globules in capillary loops resembling cryoglobulin deposits along with membranoproliferative patterns but no crescents. (Figure 1). Immunofluorescence revealed predominant IgM and kappa (Figures 2, 3). His qualitative assay for cryoglobulins became positive. His bone marrow biopsy and contrast CT of the chest, abdomen, and pelvis were normal.

Type II cryoglobulinemic vasculitis (mixed) was made as a possible diagnosis after extensive investigations. He was treated with five plasma exchanges, intravenous methylprednisolone 500 mg in three doses followed by oral prednisolone 40 mg, intravenous immunoglobulin 10 g after each cycle of plasma exchange, and intravenous rituximab 700 mg (375 mg/body surface area) in two doses two weeks apart. He underwent four hemodialysis treatments until the acute kidney injury was settled. He was discharged with mycophenolate mofetil and prednisolone. The follow-up visit showed significant improvement in proteinuria and renal function.

## Discussion

Type II cryoglobulinaemia consists of mixed cryoglobulins made up of monoclonal IgM with rheumatoid factor (RF) activity complexed with polyclonal IgG [1]. The monoclonal IgM is produced by a small, indolent B-cell or lymphoplasmacytic clone. When such a clone causes kidney injury without meeting criteria for overt lymphoma or myeloma, the condition is classified as monoclonal gammopathy of renal significance (MGRS) [1].

Pathogenetically, cryoglobulins precipitate at subphysiologic temperatures and dissolve on warming [1]. In vivo, these immune complexes deposit in small- and medium-sized vessels, activate the classical complement pathway, and cause vasculitis with consumption of complement, particularly C4. IgM monoclonal gammopathy has a high level of autoantibody activity, facilitating the formation of immune complexes, causing type II cryoglobulinemia [1].

Renal pathology most often reveals a membranoproliferative glomerulonephritis (MPGN) pattern [2] characterized by mesangial and endocapillary hypercellularity, double-contour GBM from mesangial interposition, and PAS-positive hyaline thrombi within glomerular capillaries representing precipitated cryoglobulin [2]. Immunofluorescence typically shows bright staining for monoclonal IgM (light-chain restricted, κ or λ) together with polyclonal IgG and strong C3 deposition in mesangial and capillary wall distributions. Electron microscopy often demonstrates large subendothelial electron-dense deposits with an organized microtubular or fingerprint substructure [2].

Clinical presentation can range from asymptomatic microscopic hematuria and mild proteinuria to nephrotic syndrome, chronic kidney disease, or rapidly progressive glomerulonephritis (RPGN) with crescent formation [3]. The differential diagnosis includes lupus nephritis, ANCA-associated vasculitis, and anti-GBM disease, making renal biopsy essential for definitive diagnosis.

Globally, chronic hepatitis C virus (HCV) infection is the most common cause [4]. It drives B-cell clonal expansion and monoclonal IgM production [5]. In HCV-negative cases, low-grade B-cell lymphomas, lymphoplasmacytic lymphoma, or monoclonal B-cell lymphocytosis are frequent underlying disorders.

Management aims to eliminate the pathogenic clone and control immune complex-mediated injury.

A) Clone-directed therapy: Rituximab is first-line for most B-cell clones, with or without alkylating agents (e.g., cyclophosphamide, bendamustine), depending on disease aggressiveness [6]. B) Immunosuppression: High-dose corticosteroids for rapid inflammation control, tapered as clone-directed therapy becomes effective. C) Plasmapheresis: Considered in severe, rapidly progressive, or multisystem involvement to promptly reduce circulating cryoglobulins. D) HCV-positive cases: Direct-acting antivirals in addition to immunomodulatory therapy when active vasculitis is present [7].

## Conclusions

This case highlights the importance of considering cryoglobulinemic vasculitis in the differential diagnosis of rapidly progressive glomerulonephritis and emphasizes the need for early recognition, prompting immunosuppressive therapy to prevent irreversible renal damage and improve outcome. Prognosis is determined by baseline kidney function, chronicity of histological injury, and timeliness of effective clone control.
